# Global burden of multidrug-resistant tuberculosis in children and adolescents

**DOI:** 10.1038/s41390-025-03917-1

**Published:** 2025-02-11

**Authors:** Yanyan Zhong, Huiru Xie, Fucheng Cai, Miao Liu, Hui Gan, Zhuo Tang, Yan Bai

**Affiliations:** 1 Department of Pediatrics, Huazhong University of Science and Technology Hospital, Wuhan, China; 2 Department of Pediatrics, Union Hospital, Tongji Medical College, Huazhong University of Science and Technology, Wuhan, China; 3 Department of Pediatrics, Renmin Hospital of Wuhan University, Wuhan, China; 4 Department of Allergy and Clinical Immunology, Department of Laboratory, State Key Laboratory of Respiratory Disease, National Clinical Research Center for Respiratory Disease, Guangzhou Institute of Respiratory Health, First Affiliated Hospital of Guangzhou Medical University, Guangzhou Medical University, Guangzhou, China; 5 Department of Pediatrics, Shenzhen Third People’s Hospital, Shenzhen, Guangdong Province China

## Abstract

**Objectives:**

Multidrug-resistant tuberculosis (MDR-TB) has become a major global public health issue, which has worsened over time owing to changes in disease-related trends. This study aimed to determine global trends in MDR-TB among children and adolescents for strategic health planning.

**Methods:**

A secondary analysis was performed on MDR-TB burden among children and adolescents (<20 years) at the global, regional, and national levels based on sociodemographic index (SDI) quintiles from 1990 to 2019, using the Global Burden of Disease (GBD) 2019 database.

**Results:**

In 2019, 67,710.82 (95% uncertainty interval [UI]: 38,823.61 to 110,582.03) incidents of MDR-TB were reported among children and adolescents aged <20 years worldwide. The global incidence rate has increased from 1990 to 2019, with the estimated annual percentage change (EAPC) at 4.15% (95% UI: 1.10–12.19%), particularly in low- and low-middle SDI regions. The top three highest incidence rates were observed in Southern sub-Saharan Africa, Eastern Europe, and South Asia. The mortality and disability-adjusted life years (DALYs) rates (0.62 [95% UI: 0.28–1.14] and 55.19 [95% UI: 25.24–100.74] cases per 100,000, respectively) were higher among children aged <5 years than those in older age groups in 2019.

**Conclusion:**

The global burden of MDR-TB among children and adolescents has increased from 1990 to 2019, particularly in regions with lower SDI. Extensive collaborative research and interventions are needed to mitigate this disease burden to secure the health of our future generation.

**Impact:**

- MDR-TB disease burden in children and adolescents using GBD 2019 database was analyzed.
- In total, 67710.82 MDR-TB cases were identified in children and adolescents in 2019.
- Global MDR-TB burden in children and adolescents has risen over the past 30 years.
- MDR-TB disease burden is negatively correlated with the sociodemographic index.
- MDR-TB disease burden varies across countries and age groups.

## Introduction

The emergence of multidrug-resistant tuberculosis (MDR-TB) in recent years is hampering the efforts to control TB worldwide.^1,2^ MDR-TB is characterized by resistance to both isoniazid and rifampicin and is closely linked to previous TB infections and their treatments.^3^ As MDR-TB prolongs the treatment cycles and worsens the prognosis, it has emerged as a major health and economic burden.^4^ In 2014, ~3.5% of TB cases globally, i.e., ~480,000 individuals, were classified as MDR-TB cases.^5^ China, India, Russia, and South Africa bear the highest burden of MDR-TB and collectively account for more than 60% of all cases worldwide.^6^ In addition, the South Africa region is characterized by a high prevalence of TB/human immunodeficiency virus (HIV) co-infection.^7^ Hence, there is an urgent need to develop effective MDR-TB control measures. Various trends related to MDR-TB are employed as crucial references for developing effective control strategies. The World Health Organization launched the “End TB” strategy in 2014 to achieve a 90% decline in TB incidence by 2035.^8^ To achieve this goal, it is essential to develop efficient strategies and increase the investment and commitment to MDR-TB control.

Drug-resistant *Mycobacterium tuberculosis* strains pose a serious challenge to the prevention and control efforts for TB. Notably, miRNA profiling may afford some potential biomarkers for the early diagnosis of TB.^9^ Even though children and adolescents are the most vulnerable population subgroup, the epidemiology and economic impact of MDR-TB in this section of population is poorly understood. In addition, very few studies have focused on this vulnerable group.^10^ The present study assesses the MDR-TB burden among children and adolescents for the period spanning from 1990 to 2019 using the data obtained from the Global Burden of Disease Study 2019 (GBD 2019).

## Methods

### Study data

The GBD database contains extensive data on the epidemiology and assessment of health loss from 369 diseases across 204 countries and territories, characterized by sex, GBD age groups, and GBD super-regions, countries, subnational units, and custom regions. The latest GBD database (GBD 2019) has a user-friendly approach and provides data on various factors, such as all causes, risks, impairments, injuries, and mortality rates. Consequently, it has become a valuable resource for assessing and comparing disease burden across various countries and territories.

The MDR-TB disease burden data on various aspects, such as prevalence, incidence, and DALYs, were extracted from the Global Health Data Exchange query tool of the GBD database for the period 1990–2019 for analysis (http://ghdx.healthdata.org/gbd-results-tool). Details about the study design and methods of GBD studies have been extensively described in existing literature on GBD.^11^

### Statistical analysis

This study was designed to analyze the global epidemiology and overall disease burden of MDR-TB among children and adolescents less than 20 years old based on the GBD database 2019, which is an ongoing global collaboration that uses all available epidemiological data to provide a comparative assessment of health loss from 369 diseases across 204 countries and territories.

Annual incidence cases, prevalence cases, death cases, and DALY numbers, as well as the rates of incidence, prevalence, deaths, and DALYs per 100,000 population for MDR-TB among children and adolescents were obtained from the GBD database for the period spanning 1990–2019. The resulting data were characterized based on sex, age, and location. Next, 95% uncertainty intervals (UIs) for the abovementioned metrics were calculated by fitting a randomized Markov–Chain Monte-Carlo model. DALY is currently the most widely used and representative index for the evaluation of the overall disease burden. The updated standard population age structure from GBD 2019 was used to determine the rates of incidence, prevalence, deaths, and DALYs, which were then employed to universalize the population size and age structure, referred to as number per 100,000 population. Trend rates, determined based on the estimated annual percentage change (EAPC), were employed to estimate the changing patterns of the disease burden. Further, MDR-TB burden in children and adolescents was assessed across various age groups and sex. For this study, we placed children and adolescents (<20 years) in four groups based on their age: <5 years, 5 to ≤9 years, 10 to ≤14 years, and 15 to <20 years. We analyzed the impact of development levels on MDR-TB burden using the SDI. The SDI was used to characterize the data related to per capita income at the national level, average years of education among people over 15 years of age, and total fertility rate. Based on the results obtained, we categorized the 204 countries into five SDI quintiles: high, high-middle, middle, low-middle, and low levels. This study also refined the distribution of disease burden across the GBD super-regions and 204 countries (territories).

The EAPC is a common index that is employed to reflect the temporal trend of the rate. A regression line was fitted to the natural logarithm of the rate. The EAPC and its 95% uncertainty interval (UI) were estimated using a linear regression model. The trends were assessed based on the EAPC value: if both the EAPC and 95% UI > 0, an increasing trend is suggested; however, if the EAPC and 95% UI < 0, a decreasing trend is suggested. Pearson correlation analysis was performed to analyze the correlation between the disease burden of MDR-TB and SDI.

This study utilizes data from the Global Burden of Disease (GBD) 2019 database, which is publicly available and de-identified. Since the data used in this analysis are aggregated, and anonymized, and do not involve any direct interaction with individual participants, ethical approval and informed consent were not required for this research.

All data management and statistical analyses were performed using R 4.1.0 and R studio (version 2023.09.0 + 463) and the figures were created using GraphPad Prism (version 9). All statistical assessments were two-sided, with a significance level of *P* < 0.05.

## Results

### Global trends regarding MDR-TB disease burden among children and adolescents

In 2019, a total of 67,710.82 (95% UI: 38823.61–110582.03) cases of MDR-TB in children and adolescents were recorded worldwide. A total of 95,646.65 (95% UI: 54014.64–154776.51) cases of MDR-TB were identified in 2019, resulting in 7061.39 deaths (95% UI: 3180.92–12526.53) and 611246.97 DALYs (95% UI: 283570.45–1069494.83) (Table 1).

**Table 1:** Global incidence, prevalence, death, DALYs of children and adolescents MDR-TB in 1990&2019 and trend from 1990 to 2019.

Disease burden	1990		2019		1990–2019
	Number (95% UI)	Rate/100000 (95% UI)	Number (95% UI)	Rate/100000 (95% UI)	EAPC of Rate (%,95% UI)
Incidence	11,589.03 (5069.48,25587.99)	0.51 (0.22,1.12)	67,710.82 (38823.61,110582.03)	2.63 (1.51,4.29)	4.15 (1.10,12.19)
Prevalence	13,076.98 (5852.52,28485.86)	0.58 (0.26,1.25)	95,646.65 (54014.64,154776.51)	3.71 (2.09,6.00)	5.45 (1.64,14.74)
Death	2501.04 (846.41,5876.59)	0.11 (0.04,0.26)	7061.39 (3180.92,12526.53)	0.27 (0.12,0.49)	1.49 (0.13,4.98)
DALYs	216,572.59 (73529.24,503764.06)	9.53 (3.23,22.16)	611,246.97 (283570.45,1069494.83)	23.70 (10.99,41.46)	1.49 (0.14,4.98)

*DALYs* disability-adjusted life years, *MDR‐TB* multidrug-resistant tuberculosis.

^a^Result after rounding to 2 decimal places.

The incidence, prevalence, mortality, and DALY rates in 2019 were 2.63 (95% UI: 1.51–4.29), 3.71 (95% UI: 2.09–6.00), 0.27 (95% UI: 0.12–0.49), and 23.7 (95% UI: 10.99–41.46) per 100,000 children and adolescents (<20 years of age). For girls, the incidence, prevalence, mortality, and DALY rates were 3.04 (95% UI: 1.73–4.95), 4.88 (95% UI: 2.68–8.08), 0.29 (95% UI: 0.13–0.53), and 25.32 (95% UI: 11.79–45.67) per 100,000 girls. These values are higher than those for boys. Notably, the EAPC of incidence, prevalence, mortality, and DALY rates for the period 1990–2019 were higher for girls than they were for boys (Table S1).

The mortality and DALY rates in 2019 were higher for children <5 years of age than they were for older age groups, which were 0.62 (95% UI: 0.28–1.14) and 55.19 (95% UI: 25.24–100.74) per 100,000 individuals, respectively (Table S1). The age group of 15–19 years demonstrated a significantly higher incidence rate (5.47 [95% UI: 2.64–10.17] per 100,000) and a higher prevalence rate (5.18 [95% UI: 2.35–9.48] per 100,000) than those shown by the other age groups (Table S1).

**Fig. 1:**
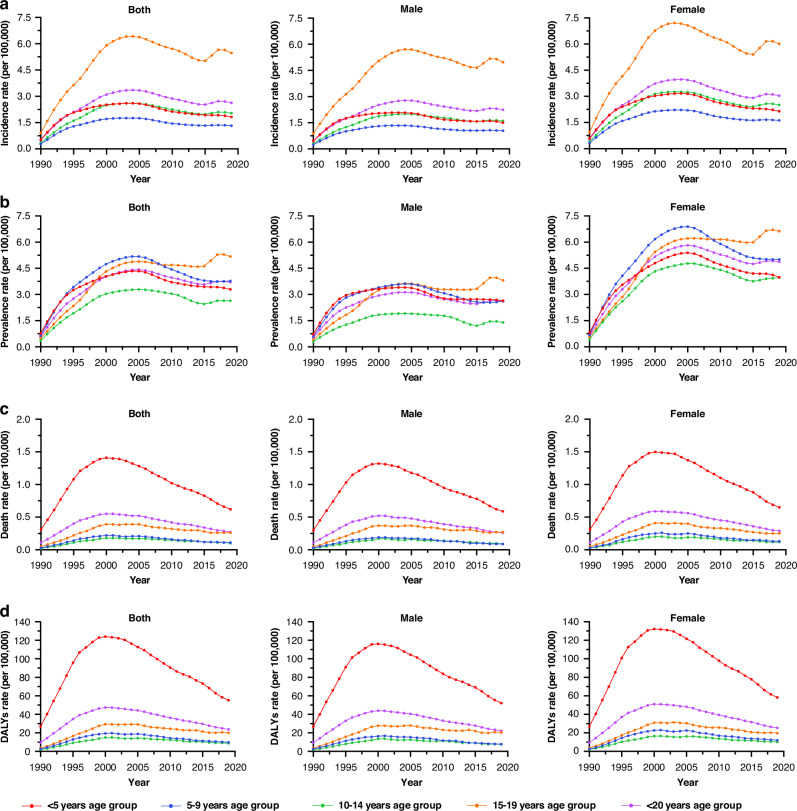
Trend for MDR-TB-related burden in children and adolescents from 1990 to 2019 based on age and sex groups. Incidence rate (**a**), prevalence rate (**b**), death rate (**c**), DALY rate (**d**); MDR‐TB multidrug-resistant tuberculosis, DALY disability-adjusted life year.

The EAPC for the rate of incidence, prevalence, death, and DALYs were 4.15% (95% UI: 1.10–12.19%), 5.45% (95% UI: 1.64–14.74%), 1.49% (95% UI: 0.13–4.98%), and 1.49% (95% UI: 0.14–4.98%), respectively, for the period spanning from 1990 to 2019 (Table 1). A steady increase in incidence rates was observed from 1990 to 2005, followed by a decline from 2005 to 2015. However, a slight rebound has been noted in the past 5 years. The 15–19 year age group demonstrated a significantly higher incidence rate compared to that exhibited by the other age groups (Fig. 1a, Table S2A). The prevalence rates steadily increased from 1990 to 2005, followed by a decline from 2005 to 2015. They have remained relatively stable between 2016 and 2019. The prevalence rates for girls of all age groups were higher than they were for boys (Fig. 1b, Table S2B). The overall mortality rate increased from 0.11 (95% UI: 0.04–0.26) per 100,000 in 1990 to 0.55 (95% UI: 0.24–0.98) per 100,000 in 2001, followed by a gradual decrease to 0.27 (95% UI: 0.12–0.49) per 100,000 in 2019. Notably, the <5-year age group exhibited a higher mortality burden than that shown by the other age groups for the period 1990–2019 (Fig. 1c, Table S2C). The DALY rate trend followed a similar pattern to mortality rates, with the <5-year age group exhibiting higher DALY rates compared to that demonstrated by other age groups during 1990–2019, for both boys and girls (Fig. 1d, Table S2D).

### MDR-TB disease burden among children and adolescents in GBD super-regions

The MDR-TB burden among children and adolescents varies in GBD super-regions. The top three highest incidence rates in 2019 were recorded in Southern sub-Saharan Africa, Eastern Europe, and South Asia [12.95(95% UI: 6.12–26.95), 5.02(95% UI: 2.99–7.79), and 5.01(95% UI: 1.69–10.85) per 100,000, respectively]. The top three highest rates of deaths and DALYs in 2019 were recorded in Southern, Central, and Eastern sub-Saharan Africa (Table S3).

Between 1990 and 2019, the incidence rate was found to decrease in East Asia, Caribbean, High-income Asia Pacific, and High-income North America. In contrast, the incidence rate increased in the remaining regions, especially in Oceania, Central Asia, and South Asia, which recorded EAPC increases of 70.28% (95% UI: 13.25–408.71%), 62.12% (95% UI: 20.21–206.30%), and 36.81% (95% UI: 6.79–180.78%), respectively (Table S3A). The death rate was observed to decrease in East Asia, High-income Asia Pacific, and High-income North America, while it was nearly zero in Central Europe, Southern Latin America, Western Europe, High-income North America, Australasia, and High-income Asia Pacific. However, a consistent increase in the death rate was observed in the remaining regions and the highest increases were recorded in Oceania, Central Asia, and South Asia, with EAPC increases of 35.31% (95% UI: 5.85–251.29%), 19.01% (95% UI: 6.62–65.14%), and 8.60% (95% UI: 1.23–46.11%) respectively (Table S3B). Overall, the MDR-TB death rate is negatively associated with SDI levels, although the death rate varies among the GBD super-regions at the same SDI level (Table S3B). The DALY rate and trend in the GBD super-regions are similar to their death burden (Table S3C).

**Fig. 2:**
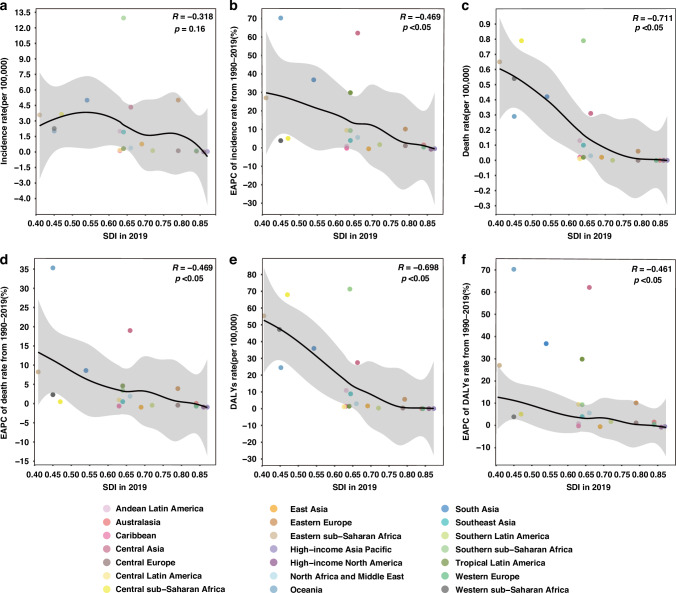
MDR-TB-related burden in children and adolescents in 2019 and trend from 1990 to 2019 in GBD super-regions with different SDI levels. Incidence rate (**a**), EAPC of incidence rate (**b**), death rate (**c**), EAPC of death rate (**d**), DALY rate (**e**), and EAPC of DALY rate (**f**). Correlation was calculated with Pearson correlation analysis. MDR‐TB multidrug-resistant tuberculosis, GBD global burden of disease, SDI sociodemographic index, DALY disability-adjusted life years]

We further explored the MDR-TB disease burden and trends related to SDI levels in the GBD super-regions. A negative correlation was found between the incidence rate of MDR-TB and SDI (*R* = −0.318, *P* = 0.16) in 2019, although the Southern sub-Saharan Africa had an expected high incidence rate at 12.95 (95% UI: 6.12–26.95) per 100,000 population and an SDI of 0.64 in 2019 (Fig. 2a). The EAPC of the MDR-TB incidence rate from 1990 to 2019 showed a significant negative correlation with the SDI level (*R* = −0.469, *P* < 0.05, Fig. 2b). The death rate in 2019 (*R* = −0.711, *P* < 0.05) and the EAPC of the death rate from 1990 to 2019 (*R* = −0.469, *P* < 0.05) exhibited a significant negative correlation with the SDI level of 2019 (Fig. 2c, d). In addition, the DALY rate in 2019 (*R* = −0.698, *P* < 0.05) and the EAPC of the DALY rate from 1990 to 2019 (*R* = −0.461, *P* < 0.05) showed a significant negative correlation with the SDI level of 2019 (Fig. 2e, f).

**Fig. 3:**
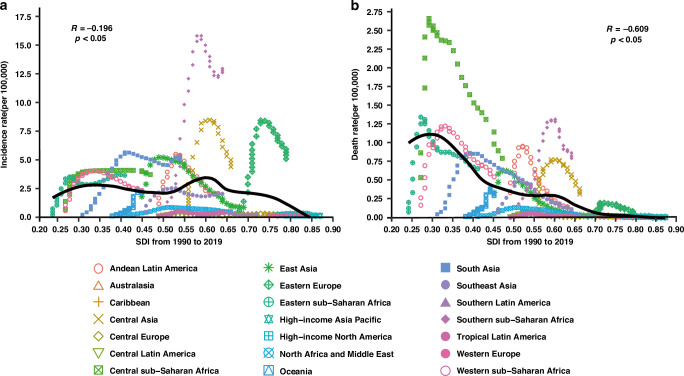
MDR-TB-related incidence and death rate in children and adolescents from 1990 to 2019 in GBD super-regions with different SDI levels. Incidence (**a**) and death (**b**) rate of children and adolescents with MDR-TB. Each point in a line represents 1 year, starting from 1990 and ending in 2019. Solid black line shows expected values across the spectrum of the SDI. MDR‐TB multidrug-resistant tuberculosis, GBD global burden of disease, SDI sociodemographic index.

To understand variations in disease burden in the GBD super-regions across different SDIs, the correlation between SDIs and MDR-TB incidence and death rates in the super-regions from 1990 to 2019 was analyzed. Overall, the MDR-TB incidence rate had a significant negative correlation with SDIs (*R* = −0.196, *P* < 0.05). For SDIs < 0.7, the incidence rate decreased with increasing SDIs in most of the GBD super-regions, except for Southern sub-Saharan Africa and Central Asia. For SDIs >0.7, the incidence rate of MDR-TB is close to 0 in most regions, except for Eastern Europe. The level of SDIs in the Eastern Europe region gradually changed from 0.68 in 1990 to 0.79 in 2019. The incidence rate rose rapidly from 0.45 (95% UI: 0.17–1.14) per 100,000 in 1990 to 8.39 (95% UI: 4.70–13.38) per 100,000 in 2005, followed by a gradual decrease to 5.02 (95% UI: 2.99–7.79) per 100,000 in 2019 (Fig. 3a, Table S4). A significant negative correlation was observed between the MDR-TB death rate and SDIs (*R* = -0.609, *P* < 0.05). However, the death rate in Southern and Central sub-Saharan African regions was higher than the expected SDI level (Fig. 3b, Table S4).

### Country-level MDR-TB disease burden among children and adolescents

**Fig. 4:**
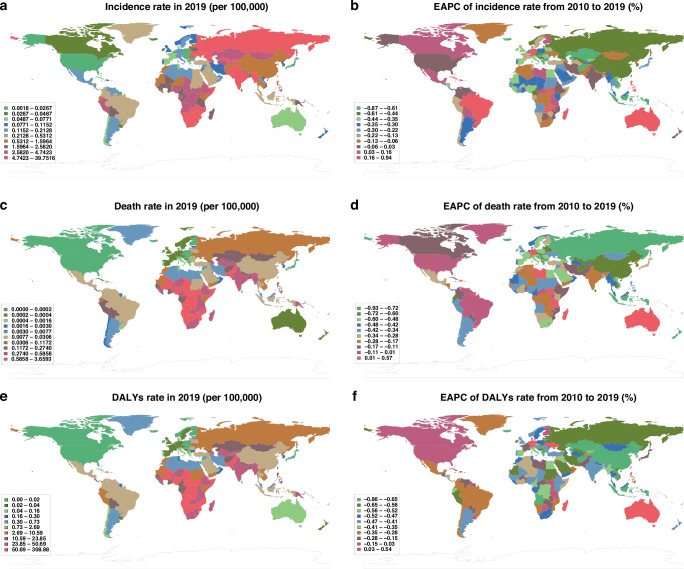
MDR-TB-related burden in children and adolescents in 2019 and EAPC trend from 2010 to 2019 in 204 countries (territories). **a** MDR‐TB incidence rate in children and adolescents in 2019 in countries/territories; **b** EAPC of children and adolescents MDR‐TB incidence rate between 2010 and 2019 in countries/territories; **c** MDR‐TB death rate among children and adolescents afflicted with the disease in 2019. **d** EAPC of children and adolescents with MDR‐TB incidence rate between 2010 and 2019. **e** MDR‐TB DALY rate among children and adolescents in 2019. **f** EAPC of MDR‐TB DALYs rate among children and adolescents with the disease between 2010 and 2019. MDR‐TB multidrug-resistant tuberculosis; EAPC estimated annual percentage change; DALY disability-adjusted life year].

The top three countries with the highest MDR-TB incidence rate in 2019 were Eswatini at 39.75 (95% UI: 9.12–97.09), Lesotho at 20.04 (95% UI: 6.66–45.24), and Namibia at 15.82 (95% UI: 6.48–31.95) per 100,000. In 2019, 49 countries had an incidence rate of <0.05 per 100,000, accounting for 24.02% of all countries (Fig. 4a, Table S5A). During 2010–2019, the top three countries with the highest EAPC of incidence rate were the Philippines at 2.06% (95% UI: −0.25% to 7.81%), Papua New Guinea at 1.66% (95% UI: -0.34% to 9.45%), and Comoros at 1.62% (95% UI: −0.44% to 11.56%). A total of 67 countries had a positive EAPC of the MDR-TB incidence rate, accounting for 32.84% (Fig. 4b, Table S5B). The MDR-TB incidence rate showed a stable decline in Guam, Hungary, Kazakhstan, Cyprus, Slovenia, and Iceland, with both EAPC and 95% UI < 0 (Fig. 4b, Table S5B).

A significantly high MDR-TB death burden among children and adolescents was observed in African countries. The top three countries with the highest death rate were Somalia, Eswatini, and Lesotho in 2019 [3.66 (95% UI: 0.86–9.61), 3.28 (95% UI: 0.74–7.30), and 2.41 (95% UI: 0.65–5.87) per 100,000, respectively]. In 2019, 92 countries had a death rate of nearly 0, accounting for 45.1% of all countries (Fig. 4c, Table S5C). During 2010–2019, the EAPC for the MDR-TB death rate was positive for 23 countries (Fig. 4d, Table S5D).

In 2019, the top three countries with the highest DALY rates for children and adolescents with MDR-TB were Somalia, Eswatini, and Lesotho [308.88 (95% UI: 72.24–811.08), 289.93 (95% UI: 63.68–652.98), and 207.26 (95% UI: 58.64–498.19) per 100,000, respectively] (Fig. 4e, Table S5E). Notably, 21 countries, accounting for 10.29% of all countries that we considered herein, recorded a stable decline in the MDR-TB DALY rate. The top three countries with the highest EAPC of the DALY rate from 2010 to 2019 were Papua New Guinea at 0.88% (95% UI: −0.54% to 6.46%), Bermuda at 0.54% (95% UI: −0.84% to 10.69%), and Dominica at 0.41% (95% UI: −0.77% to 7.44%) (Fig. 4f, Table S5F).

## Discussion

This is the first study to use data from GBD 2019 to conduct a comprehensive and in-depth investigation of the disease burden and trends of MDR-TB among children and adolescents across 204 countries or regions around the world in the past 30 years. The study showed that the global incidence and prevalence of MDR-TB among children and adolescents remained high in 2019, particularly in Southern sub-Saharan Africa, Eastern Europe, and South Asia. Death rate among children and adolescents afflicted with MDR-TB was high in sub-Saharan Africa, South Asia, and Central Asia in 2019. The global MDR-TB burden among children and adolescents increased from 1990 to 2019, especially in Oceania, Central Asia, and South Asia. The 15–19-year age group reported a significantly higher incidence rate than that for the other given age groups. The <5-year age group had higher mortality rates than that of the other given age groups for the period 1990–2019. Our findings indicate that public policies and community programs focusing only on the prevention of MDR-TB among children and adolescents are insufficient. Hence, there is an urgent need to develop region-specific and age-tailored prevention strategies for MDR-TB.

Previous works based on GBD 2015^12^ and 2017^13^ estimated the global burden of MDR-TB for the whole population. They reported a worldwide decrease in MDR-TB incidence, prevalence, death, and DALYs. Popularization of Bacillus Calmette–Guerin (BCG) vaccination, widespread use of tuberculosis preventive treatment (TPT), and continuous improvement in diagnosis methods and treatment strategy have led to a significant decrease in the burden of MDR-TB-related diseases in the general population since 2000. However, the disease burden in children and adolescents has still not been effectively controlled, suggesting that more concentrated and targeted efforts are needed for the prevention and treatment of MDR-TB to effectively control its spread and related deaths in children and adolescents. A study in Northern Ethiopia reported that the sharing of drink and food materials, active TB contact, a low body mass index, and an HIV status are statistically significant predisposing factors for rifampicin-resistant TB.^7^ Hence, it can be suggested that controlling risk factors may reduce the occurrence of MDR-TB.

BCG vaccination is effective in preventing pulmonary tuberculosis.^14^ It may also prevent the occurrence of miliary and meningeal tuberculosis. However, its efficacy varies in real-world settings. A child develops MDR-TB mainly by inhaling *M. tuberculosis* resistant to isoniazid and rifampicin.^15^ Hence, there is an urgent need to develop suitable TPT for children with household contacts of MDR-TB.^16^ The WHO’s End TB Strategy recommends providing TPT to all household contacts, regardless of age.^17,18^ In addition, school-based active case-finding and provision of TPT can reduce TB transmission among adolescents in both low-prevalence and high-prevalence regions.^19,20^ Novel vaccines and TPT may help in preventing MDR-TB transmission among children and adolescents.

Despite a decline in mortality rates related to MDR-TB among children and adolescents, approximately 7061.39 (95% UI: 3180.92–12526.53) cases were still recorded worldwide in 2019. Our study shows that the death rate was the highest among children <5 years of age, as younger children are at a greater risk of tuberculous meningitis and acute pulmonary miliary tuberculosis.^21,22^ However, further study is needed to investigate this topic. A meta-analysis^23^ on 1343 children reported an overall pooled treatment success of 77.0% (95% CI: 69.0–85.0). Pooled treatment had a lower success rate in developing countries (73.0%, 95% CI: 63.0–83.0) compared to that in developed countries (87.0%, 95% CI: 81.0–94.0). For children with MDR tuberculosis, high cure rates can be achieved by using tailored regimens using second-line drugs in clinical trial settings. However, children <5 years of age face a significantly higher risk of death or treatment failure if they have a severe infection, are underweight, and have HIV infection.^24,25^ Limitations of diagnostic assays, shortage of drugs at health centers, and a shortage of clinicians trained in childhood tuberculosis are responsible for the delay in diagnostic evaluation and initial treatment of the disease in low-SDI regions.^26^ Although nutritional support is acknowledged as a major intervention in TB programs, it is seldom prioritized for action.^27^ To lower the death rate among children <5 years of age, it is necessary to conduct a rapid diagnosis of MDR-TB by ensuring a steady supply of second-line anti-tuberculosis drugs, providing adequate nutritional support, and managing various comorbidities.^28^

Our study results showed that MDR-TB disease burden among children and adolescents is closely linked to the SDI level. Southern sub-Saharan Africa and Central Asia have a higher tuberculosis burden than expected, owing to their current sociodemographic development level. Southern sub-Saharan African regions face serious healthcare challenges owing to the HIV epidemic and MDR-TB co-infection. A previous meta-analysis^29^ showed that the odds of MDR-TB among HIV-positive cases are 1.42 times higher than that among HIV-negative cases. Surprisingly, several countries with a low incidence of MDR-TB among children and adolescents reported increasing trends for DALYs from 2010 to 2019, such as Papua New Guinea, Bermuda, Dominica, and Australia. Indeed, Papua New Guinea has a high TB burden, making cross-border TB management in Australia a challenging task.^30^ Therefore, the importance of pre-immigration tuberculosis screening should be emphasized.^31^ Low- and low-middle SDI regions face a significantly high burden of MDR-TB among children and adolescents. Hence, these regions should allocate more resources for handling MDR-TB in children and adolescents. However, some middle- to high-SDI regions are also grappling with new challenges, such as HIV infection and pre-immigration screening during MDR-TB prevention and management.

### Limitations

This study has some limitations as well. First, the accuracy of the GBD estimate greatly depends on the availability and quality of data. For example, in countries without vital registration data, tuberculosis mortality is estimated using verbal autopsy studies, which proved to be credible and sensitive. Second, our analysis of the relationship between SDI and MDR-TB incidence, prevalence, and mortality should be interpreted as an average historical correlation rather than a causal relationship. Social heterogeneity within countries should be considered to enhance the applicability of SDI. Third, risk factors and clinical information for MDR-TB were not available, making it difficult to fully investigate the changing trends. However, annual updating of the GBD database will allow for the refinement of the methodology and more accurate estimation of the MDR-TB burden among children and adolescents as data quality continues to improve.

## Conclusion

Our study showed that the MDR-TB burden among children and adolescents has increased globally over the past three decades, which is closely linked to the SDI level. Southern sub-Saharan Africa and Central Asia had a higher tuberculosis burden than expected. In addition, the global age distribution of tuberculosis cases and deaths showed a notable difference. Cases were highest among 15–19-year-old adolescents, while deaths were highest among <5-year-old children. Further work is needed to investigate the reasons for the age and area differences. There is also an urgent need to develop effective policies and interventions to reduce the burden of MDR-TB among children and adolescents in high-prevalence regions, such as Southern sub-Saharan Africa, Eastern Europe, and South Asia. Policymakers and health care providers need to prioritize BCG vaccination, rapid diagnosis, drug supply, and equal health care access to meet the “End TB” target by 2030.

## Supplementary information

Table S1A

Table S1B

Table S1C

Table S1D

Table S2A

Table S2B

Table S2C

Table S2D

Table S3A

Table S3B

Table S3C

Table S4

Table S5A

Table S5B

Table S5C

Table S5D

Table S5E

Table S5F

## Data Availability

The datasets analyzed during the current study are available from the corresponding author upon reasonable request.
